# Increased Circulating Levels of PCSK9 and Pro-Atherogenic Lipoprotein Profile in Pregnant Women with Maternal Supraphysiological Hypercholesterolemia

**DOI:** 10.3390/antiox11050869

**Published:** 2022-04-28

**Authors:** Claudette Cantin, María Jesús Garchitorena, Rodrigo Escalona, Jorge A. Carvajal, Sebastián E. Illanes, Jaime Gutierrez, Andrea Leiva

**Affiliations:** 1 School of Medicine, Faculty of Medicine, Pontificia Universidad Católica de Chile, Santiago 8331150, Chile; clcantin@uc.cl (C.C.); mjgarchitorena@uc.cl (M.J.G.); jcarva@med.puc.cl (J.A.C.); 2 School of Medical Technology, Faculty of Medicine and Sciences, Universidad San Sebastián, Santiago 7510157, Chile; rescalonar@correo.uss.cl (R.E.); jaime.gutierrez@uss.cl (J.G.); 3 Faculty of Medicine, Universidad de los Andes, Santiago 7550000, Chile; sillanes@uandes.cl

**Keywords:** pregnancy, low-density lipoproteins, PCSK9, atherogenic profile

## Abstract

Maternal physiological hypercholesterolemia (MPH) occurs during pregnancy to assure fetal development. Some pregnant women develop maternal supraphysiological hypercholesterolemia (MSPH) characterized by increased levels of low-density lipoprotein (LDL). We aim to determine if proprotein convertase subtilisin/kexin type 9 (PCSK9) levels (a protein that regulate the availability of LDL receptor in the cells surface), as well as the composition and function of LDL, are modulated in MSPH women. This study included 122 pregnant women. Maternal total cholesterol (TC), LDL, triglycerides and PCSK9 increased from first (T1) to third trimester (T3) in MPH women. At T3, maternal TC, LDL, PCSK9 and placental abundances of PCSK9 were significantly higher in MPSH compared to MPH. Circulating PCSK9 levels were correlated with LDL at T3. In MSPH women, the levels of lipid peroxidation and oxidized LDL were significantly higher compared to MPH. LDL isolated from MSPH women presented significantly higher triglycerides and ApoB but lower levels of ApoAI compared to MPH. The formation of conjugated dienes was earlier in LDL from MSPH and in endothelial cells incubated with these LDLs; the levels of reactive oxygen species were significantly higher compared to LDL from MPH. We conclude that increased maternal PCSK9 would contribute to the maternal elevated levels of pro-atherogenic LDL in MSPH, which could eventually be related to maternal vascular dysfunction.

## 1. Introduction

Increased maternal plasma cholesterol levels during pregnancy is a physiological condition that results from the requirements of the growing fetus and is referred as maternal physiological hypercholesterolemia (MPH, maternal total cholesterol (TC) at term ≤ 280 mg/dL) [1,2]. In some pregnant women and for unknown mechanisms, maternal plasma cholesterol increases over this physiological level (i.e., maternal supraphysiological hypercholesterolemia, MSPH) [3,4]. MSPH is determined in women with TC levels above a cut-off value of 280–300 mg/dL at terms of pregnancy or above the 75th percentile for the different trimesters of pregnancy [1,2,3,4,5,6]. Interestingly, in MSPH, the maternal levels of low-density lipoproteins (LDL) increased, without changes in high-density lipoprotein (HDL) or triglyceride levels [1,2,3,4,5,6]. This maternal condition is associated with the endothelial dysfunction of the macrovascular and the microvascular vessels of the placenta [4,7,8], altered the traffic and content of cholesterol in placental trophoblasts [9] and affected the development of atherosclerosis in the fetal aorta [1] and in the aorta of children and adolescents [2], as well as with major cardiovascular risk in adults [10] of offspring developed in an MSPH pregnancy. This information suggests that MSPH could be related with the development of cardiovascular disease in offspring later in life [2,6,11,12]. However, the effects of MSPH in women exposed to these increased levels of lipids are unknown. Despite the increased maternal concentrations of TC and LDL, no studies addressing any possible factors associated with this phenomenon have been reported. Furthermore, the functional characteristics of these maternal LDLs are unknown.

Circulating LDL levels are typically determined by the availability of LDL receptor (LDLR) on the cells relevant for cholesterol homeostasis [13]. Proprotein convertase subtilisin/kexin type 9 (PCSK9) is a serine proteinase that modulates cholesterol metabolism through the lysosomal degradation of LDLR, leading to decreased levels of the receptor in the cellular surface. Therefore, the hepatic levels of PCSK9 positively correlates with circulating levels of LDL [14,15]. Interestingly, the plasma determination of PCSK9 indirectly reflexes the levels and activity of this protein in the liver, which have been used as a suitable tool in research and in clinical practice [16]. The relevance of PCSK9 in the regulation of plasma LDL levels in non-pregnant population is that the inhibitors of the protein are considered as a therapeutic target with the potential of statins [14]. Several metabolic parameters, including age, sex, body mass index, menopausal status, pregnancy and circulating levels of lipids and glucose are correlated with PCSK9 levels [14,17,18]. In pregnancy, it has been described that PCSK9 levels are increased compared to non-pregnant women [18]. However, whether the levels of PCSK9 are differentially modulated in MPH and MSPH pregnancies is currently unknown.

In addition to the increased levels of TC and LDL in a physiological pregnancy, changes towards a pro-atherogenic profile of maternal lipoproteins have been reported during pregnancy. In physiological pregnancies, the increase in lipids levels occurs mainly during the second and third trimesters. In the maternal liver, the synthesis of lipoproteins enriched in triglycerides (VLDL and LDL) increased due to the augmented hepatic synthesis of apolipoprotein B (the principal apolipoprotein and precursor of these lipoproteins) and by the reduced activity of lipoprotein lipase (LPL), which is responsible for the plasmatic clearance of VLDL, in a process regulated by insulin-resistance [19] and increased estrogen levels that are characteristic of pregnancy [20]. Increased levels of LDL are also related with pregnancy-dependent increased activity of cholesteryl ester transfer protein (CETP), an enzyme that facilitates the exchange of cholesteryl ester (CE) for triacylglycerol between HDL and non-HDL lipoproteins as VLDL and LDL [21]. Because of the action of this enzyme, LDLs are enriched in CE and HDLs in triglycerides. Therefore, pregnancy associates with changes in the lipoprotein profile, being more abundant in maternal circulation in smaller and denser LDL (sdLDL) compared to LDLs of age-matched non-pregnant women [22,23,24,25,26,27]. These sdLDL are more susceptible to oxidation than the larger subfractions considering not only the composition but also that the circulation time of sdLDL is longer than those of large LDL, increasing the probability of atherogenic modifications such as oxidation [26]. In fact, at the end of pregnancy, there are increased levels of maternal oxidized LDL (oxLDL), one of the major factors involved in the development of endothelial dysfunction and atherosclerosis [26]. By decades, oxLDLs have been named by clinicians as sdLDL, and currently, both oxLDL and sdLDL are considered to be independently associated with cardiovascular events [26]. Despite this information, it is unknown whether the proatherogenic characteristics of LDLs are comparable to those of MPH or whether they are exacerbated in MSPH pregnancies.

Considering that increased levels of circulating LDL have been described in MSPH women, we aimed to determine if PCSK9 levels are modulated in these women, as well as the composition and function of its LDLs.

## 2. Materials and Methods

### 2.1. Study Groups

Maternal and umbilical cord blood was obtained from 122 pregnant women from the Hospital Clínico UC-CHRISTUS (HCUC) and Clínica Dávila, Chile. The investigation was conformed to the principles outlined in the Declaration of Helsinki. The protocol was approved by the Ethics Comitte of the Faculty of Medicine of the Pontificia Universidad Católica de Chile (PUC) and Clínica Dávila (ID 180810004). Informed consent and clinical data from patients were obtained as previously described [9]. Maternal (i.e., age, height, weight, blood pressure and glucose levels) and neonatal characteristics (i.e., sex, gestational age, weight, height and APGAR score) were obtained from the clinical records by the medical staff. In maternal blood, the levels of TC, HDL, LDL and triglycerides were determined at first (T1, *n* = 16) or third (T3, *n* = 106) trimester of pregnancy.

In the NPH group, women with TC < 280 mg/dL at the term of pregnancy (*n* = 16 for T1 and *n* = 44 for T3) were included. The women with TC ≥ 280 md/dL were included in the MSPH group (*n* = 43 at T3). The cut-off value for MSPH correspond to TC levels, and fetoplacental endothelial and vascular dysfunction have been described [1,2,4,5,8,27,28]. Exclusion criteria included the following: maternal obesity at term of pregnancy, diabetes, preeclampsia, intrauterine growth restriction, fetal malformations and other maternal pathologies. In addition, some parameters were also determined in a group of pregnant women with gestational diabetes mellitus (GDM, *n* = 19). GDM was diagnosed between the 24th and 28th week of gestation with one of two values over the following cut-off points: fasting glycemia ≥ 100 mg/dL (5.6 mM) or ≥140 mg/dL (7.8 mM) at two hours after a 75 g glucose load [29].

### 2.2. Determination of Maternal Cholesterol and Triglyceride Levels

Maternal al neonatal lipid profile (TC, HDL, LDL and triglycerides) was assayed in serum from brachial venous blood or umbilical cord blood as described [8]. Enzymatic-colorimetric assays were performed in the Clinical Laboratory of the HCUC, as previously reported [7,8].

### 2.3. Determination of PCSK9 in Serum

PCSK9 levels were determined in maternal serum by ELISA (human PCSK9 Quantikine ELISA kit DPC900, R&D Systems, Minneapolis, MN, USA). Aliquots of serum were frozen (−80 °C) after centrifugation of maternal blood, and the determination of PCSK9 was performed in two replicated assays.

### 2.4. LDL Isolation

LDLs from the women of this study were isolated by ultracentrifugation as described. [9]. Briefly, sucrose (final concentration: 10%), EDTA (10 mmol/L, pH 7.4), aprotinin (2 μg/mL) and phenylmethylsulfonyl fluoride (PMSF, 1 mmol/L) were added to the fresh serum. The density of the mixed serum was adjusted to 1.24 g/mL with potassium bromide (KBr). A gradient was generated by the addition of PBS (3.3 mL, density: 1.006 g/mL) to the serum (1.7 mL, density: 1.24 g/mL). The gradients were ultracentrifugated at 287,000 RCF, 15 °C and 4 h (rotor Beckman SW55Ti). After centrifugation, the bands corresponding to LDL and HDL were collected from the gradient. Isolated lipoproteins were dialyzed in saline solution (mmol/L 150 NaCl, 0.34 EDTA, pH 7.4, 4 °C, 48 h) and later stored at 4 °C in tubes saturated with nitrogen. The protein concentration was determined as described in the Western blot section. The correct isolation of LDL was determined by SDS-PAGE separation followed by Coomassie R-250 staining and Western blot for ApoB and ApoAI.

### 2.5. Western Blot

Protein abundance was determined in isolated LDL and in homogenized placenta. Sections of placental tissue were lysed in solution 1 (mmol/L 10 EDTA, 50 Tris-HCl, pH 8.3) and the same volume of solution 2 (4% SDS, 20% glycerol, 125 mmol/L Tris/HCl, pH 6.8). The reactions were heated (50 °C, 10 min), sonicated (6 cycles, 10 s, 100 Watts, 4 °C) and spun down (15,000 RCF, 20 min) as described [9,28]. Protein content was determined with the bicinchoninic acid (micro BCA) protein assay kit (Thermo Fisher Scientific, Waltham, MA, USA).

The proteins were separated by polyacrylamide gel electrophoresis in denaturing and reducing conditions. After that, the proteins were transferred to polyvinylidene difluoride membranes and later probed with primary rabbit polyclonal anti-apolipoprotein B (ApoB), anti- apolipoprotein AI or AII (ApoAI, ApoAII) or mouse monoclonal anti- apolipoprotein E (ApoE) (Abcam, Cambridge, UK) (1:1000, 18 h, 4 °C), anti-PCSK9 (PCSK9, 1:500, 18 h, 4 °C) (Santa Cruz Biotechnology, Dallas, TX, USA) and anti-β-tubulin (1:5000, 1 h, room temperature) (Sigma-Aldrich, St. Louis, MI, USA) antibodies. After incubation, the membranes were washed and incubated with secondary antibody conjugates with horseradish peroxidase goat anti-rabbit or anti-mouse antibody (Thermo Fisher Scientific, Waltham, MA, USA) by 1 h. The membrane was washed again and the proteins were detected by enhanced chemiluminescence and quantified by densitometry.

### 2.6. Lipid Determination Assays

Total, ester and free cholesterol were determined in isolated LDL by AmplexRed cholesterol assay kit (Thermo Fisher, Waltham, MA, USA). Triglycerides were determined in isolated LDL by serum triglyceride determination kit (Sigma-Aldrich, St. Louis, MI, USA) according to the manufacturer’s instruction. Results were expressed as μM according to calibration curves.

### 2.7. TBARS Assay

Lipid peroxidation of MPH and MSPH serum was assessed by TBARS formation (TBARS assay kit, Cayman Chemical, Ann Arbor, MI, USA). Briefly, samples were incubated with 0.1 mL of SDS Solution and 4 mL of Color Reagent. After heating at 100 °C for 1 h, the samples were incubated on ice and centrifugated at 1600× *g* for 10 min. A calibration curve was prepared with malondialdehyde (MDA) standard. MDA concentration was estimated by absorbance at 532 nm. Results were expressed as μM MDA.

### 2.8. Determination of Oxidized LDL in Serum

Oxidized LDL levels were determined in maternal serum by ELISA (human oxLDL ELISA kit, Cusabio CSB-E08797h, Wuham, China). Aliquots of serum were frozen (−80 °C) after centrifugation of maternal blood, and the determination of oxLDL was performed in two replicated assays.

### 2.9. Human Umbilical Vein Endothelial Cells Culture

Human umbilical vein endothelial cells (HUVEC) were isolated by collagenase II digestion from umbilical cords obtained from normal term pregnancies at birth, as described [28]. The cells were cultured (37 °C, 5% CO_2_) in medium 199 (M199; Gibco, Waltham, MA, USA) containing 5 mmol/L D-glucose, 10% newborn calf serum (NBCS), 10% fetal calf serum (FCS) (Gibco), 3.2 mmol/L L-glutamine and 100 U/mL penicillin-streptomycin (primary culture medium, PCM) (Gibco). Twenty-four hours prior to experiments, the incubation medium was changed to 2% NBCS/FCS containing M199.

### 2.10. Intracellular Reactive Oxygen Species (ROS) Determination

Intracellular ROS levels were determined using the fluorescent dye 5-(and-6)-chloromethyl-2′,7′-dichlorodihydrofluorescein diacetate (CM-H2DCFDA, Sigma-Aldrich, St. Luois, MI, USA), as previously described [29]. Cells were exposed to LDL from MPH or MSPH women (50 μg/mL, 1 h, 37 °C) in the absence or presence of copper sulphate (CuSO_4_; 10 μM, 3 h) as a positive control. After washing, cells were incubated (30 min, 37 °C) with 10 μmol/L of CM-DCFDA, and fluorescence (λexc/λem: 485/535 nm) was determined in a Synergy H1 microplate reader. After measurements, cells were lysed with 0.5 N KOH and the proteins were determined with Bradford reagent. ROS levels were expressed as arbitrary units of fluorescence per μg of protein.

### 2.11. Conjugated Dienes Formation

Conjugated dienes formation was determined in maternal lipoproteins (50 μg/mL) incubated with CuSO4 (10 μM), measuring the absorbance at 234 nm at intervals of 5 min for 6 h and at 37 °C in a Synergy H1 microplate reader. LDL oxidation kinetics were graphed, and the lag time (T lag), maximal dienes concentration, maximal oxidation rate and maximal time of dienes formation (T max) were calculated, as described [30].

### 2.12. Statistical Analysis

Maternal and neonatal clinical data are shown as mean ± S.D., as described [9]. Results from in vitro assays are shown as mean ± S.E.M. In all the experiments, *n* indicates the number of serum samples, placentas or cell cultures used. The sample size was estimated by the mean comparison approach considering a power of 80% to detect a difference between both groups based on a two-sided alpha level of 0.05. Pearson’s or Spearman’s correlation were used to analyze LDL and PCSK9 levels. Student’s *t*-test or ANOVA were used to compare two or more groups, respectively. *p* < 0.05 was considered statistically significant. The data were analyzed by using the software GraphPad Prism 7.0 (GraphPad Software Inc., San Diego, CA, USA).

## 3. Results

### 3.1. Clinical Characteristics of the Studied Groups

This study considered 122 pregnant women without diagnosed pathologies and 19 with gestational diabetes mellitus. As described in the previous section, pregnant women with TC < 280 mg/dL at term of pregnancy were included in the MPH group (*n* = 16 for MPH-T1 and *n* = 44 for MPH-T3). The women with TC ≥ 280 md/dL were included in the MSPH group (*n* = 43, MSPH-T3). The descriptions of the maternal and offspring clinical data of the studied women are presented in Table 1. Despite weeks of gestation at which was assayed the lipid profile, no significant differences were found among women from the groups MPH-T1, MPH-T3 and MSPH-T3 (Table 1). In addition, between the groups MPH-T1, MPH-T3, MSPH-T3 and GDM, the main difference was the higher maternal age (*p* < 0.05) and the result of the OTGG test in the GDM group compared to the other groups (*p* < 0.05) (Table 1).

### 3.2. Maternal Lipids and PCSK9 Levels at First and Third Trimester of Pregnancy

**Figure 1:**
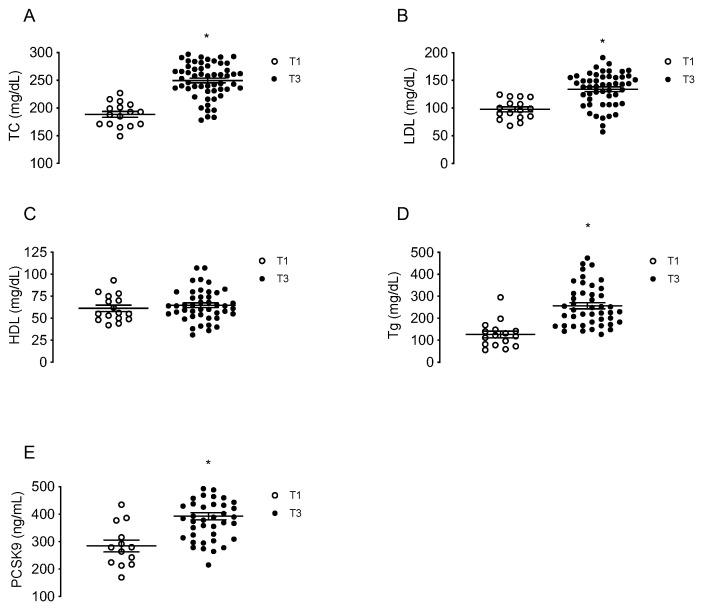
Maternal concentrations of lipids and PCSK9 at first and third trimesters of pregnancy. Total cholesterol (TC, (**A**)), LDL (**B**), HDL (**C**), triglycerides (Tg, (**D**)) and circulating levels of PCSK9 (**E**) were determined in serum from MPH pregnant women at first (T1, *n* = 16, white) and third trimesters (T3, *n* = 44, black). Values are mean ± S.E.M. * *p* < 0.05 versus values for T1.

Maternal concentrations of TC, LDL, HDL, triglycerides and circulating PCSK9 were determined in a group of MPH pregnant woman at T1 (*n* = 16) and in other groups of MPH women at T3 (*n* = 44). TC, LDL and triglycerides levels were higher at T3 compared to T1 (32.4%; 36.9% and 103%, respectively, *p* < 0.05), without changes in HDL (Figure 1A–D). The levels of circulating PCSK9 were 39.4% higher at T3 compared to T1 (*p* < 0.05) (Figure 1E).

### 3.3. Maternal Lipids and PCSK9 Levels in MPH and MSPH Pregnancies

**Figure 2:**
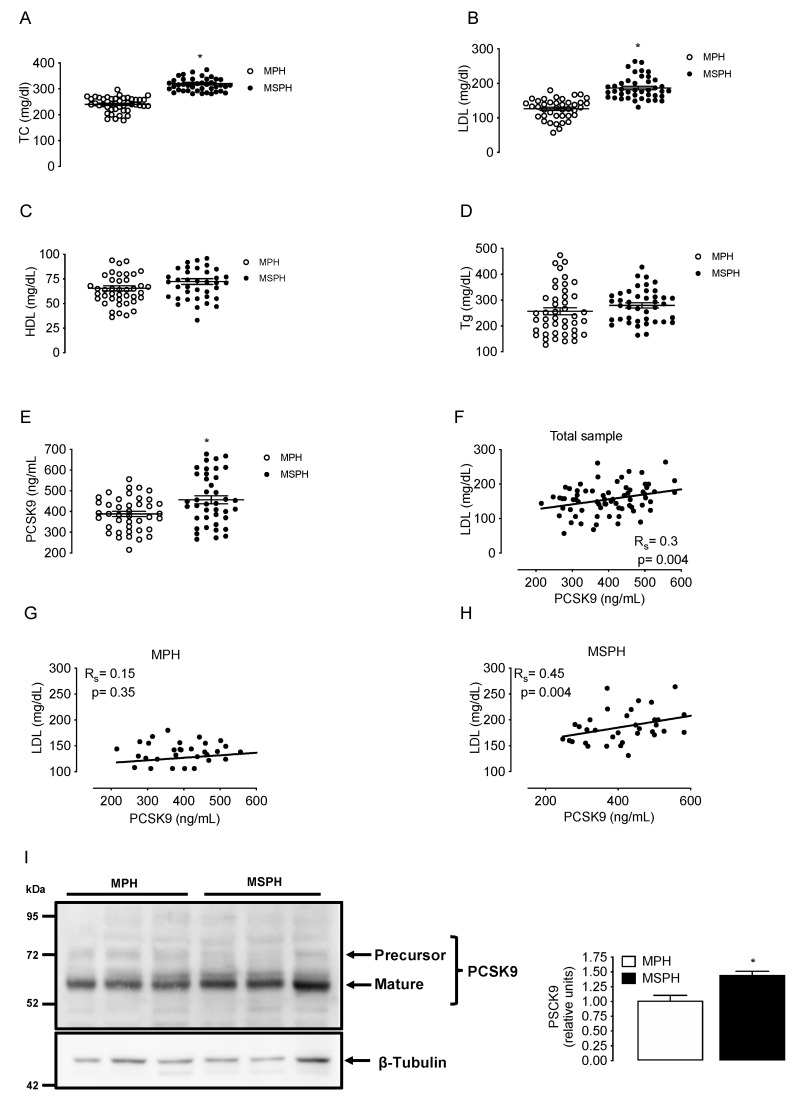
Maternal concentrations of lipids and PCSK9 in MPH and MSPH women. Total cholesterol (TC, (**A**)), LDL (**B**), HDL (**C**), triglycerides (Tg, (**D**)) and circulating levels PCSK9 (**E**) were determined in serum from MPH (*n* = 44, white) and MSPH women at T3 (*n* = 43, black). (**F**) Spearman’s correlation (R_s_) between LDL and PCSK9 levels in the entire sample (MPH + MPSH, *n* = 87). (**G**) Spearman’s correlation between LDL and PCSK9 levels in the MPH group. (**H**) Spearman’s correlation between LDL and PCSK9 levels in the MSPH group. (**I**) Representative Western blot showing the protein abundance of PCSK9 determined in the extract of whole placenta. Blot quantification is shown in the bar graph (*n* = 6 per group). Values are mean ± S.E.M. * *p* < 0.05 versus values for MPH.

Maternal concentrations of TC, LDL, HDL, triglycerides and circulating PCSK9 were determined in a group of MPH pregnant woman (*n* = 44) and in a group of MSPH women (*n* = 43) at T3. TC and LDL levels were higher in MSPH compared to MPH women (30.4% and 48%, respectively, *p* < 0.05) without changes in HDL or triglycerides (Figure 2A–D). The levels of circulating PCSK9 were 18.7% higher in MSPH compared to MPH women (*p* < 0.05) (Figure 2E). 

The correlation between LDL and PCSK9 was firstly assayed in the entire sample (MPH + MPSH, *n* = 87). The correlation was positive, with an R value (Spearman’s) of 0.3 (*p* = 0.004) (Figure 2F). After that, the correlation with LDL was assayed by separation in MSPH and MSPH women. The correlation between LDL and PCSK9 in the MPH group was not significative (Figure 2G). However, the correlation between LDL and PCSK9 in the MSPH group was positive, with an R value (Spearman) of 0.45 (*p* = 0.004) (Figure 2H). 

To determine tissue levels of PCSK9, the protein was determined in extracts of whole placenta (*n* = 6 per group). The abundance of PCSK9 was 43% higher in MSPH compared to MPH placentas (*p* < 0.05) (Figure 2I).

**Figure 3:**
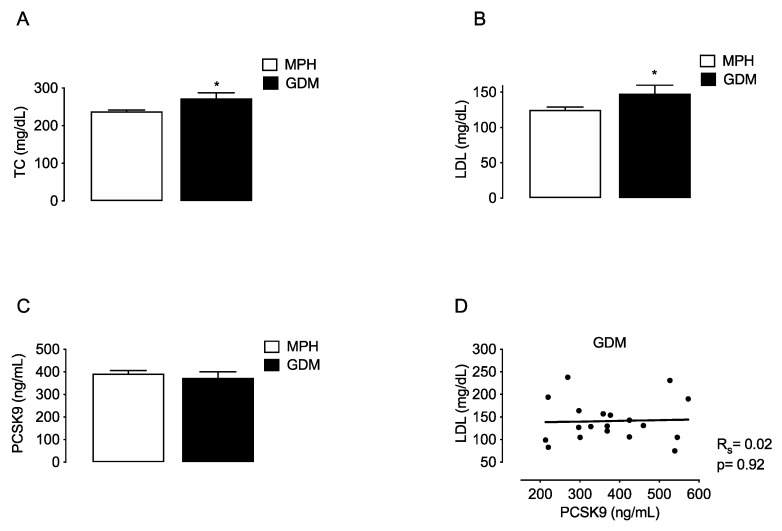
Maternal concentrations of lipids and PCSK9 in MPH and GDM women. Total cholesterol (TC, (**A**)), LDL (**B**), HDL (**C**) and circulating levels PCSK9 (**C**) were determined in serum from MPH women (*n* = 44, white) and women with gestational diabetes mellitus women at T3 (GDM, *n* = 19, black). (**D**) Spearman’s correlation (R_s_) between LDL and PCSK9 levels in the the GDM group. Values are mean ± S.E.M. * *p* < 0.05 versus values for MPH.

To compare if changes in PCSK9 are associated with MSPH or with other maternal conditions that also relate with increased TC and LDL levels, the lipids and PCSK9 were evaluated in a group of women with gestational diabetes mellitus (GDM, *n* = 19). TC and LDL levels were higher in GDM compared to MPH women (14.7% and 18.4%, respectively, *p* < 0.05) (Figure 3A,B). Circulating PCSK9 levels were similar in GDM compared to MPH women (Figure 3C), and no correlation between LDL and PCSK9 was found in the GDM group (Figure 3D). 

### 3.4. Composition of Maternal LDL from MPH and MSPH Pregnancies

**Figure 4:**
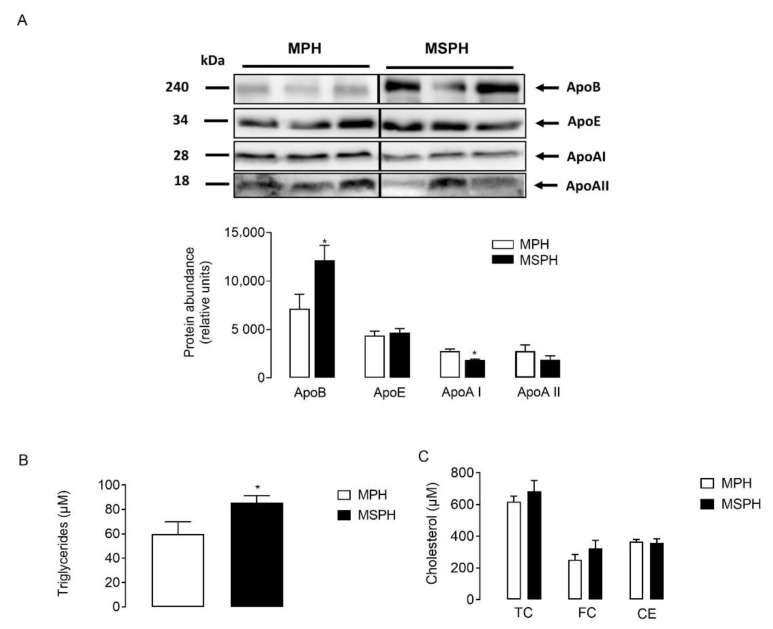
Composition of maternal LDL from MPH and MSPH women. (**A**) Representative Western blot showing the protein abundance of Apolipoprotein B (ApoB), AI (ApoAI) and E (ApoE), determined in LDL isolated from MPH (white) and MSPH (black) maternal serum. Blot quantification is shown in the bar graph (*n* = 7 per group). Equal quantities of proteins (30 ug) were loaded for each sample. (**B**) Triglycerides levels determined in LDL isolated from MPH (white) and MSPH (black) maternal serum. (**C**) Levels of cholesterol: total (TC), free (FC) and ester (CE) in LDL isolated from MPH (white) and MSPH (black) maternal serum. Values are mean ± S.E.M. * *p* < 0.05 versus values for MPH.

LDLs were isolated from MPH and MSPH serum (*n* = 7 per group) and equal protein mass was separated by electrophoresis. The protein abundance of ApoE and ApoAII was comparable between groups (Figure 4A). However, the levels of ApoB were higher in LDL from MSPH women (*p* < 0.05) (Figure 4A). Although the levels of ApoAI were significatively lower in LDL compared to HDL after ultracentifugation (data not shown), the protein was quantifiable in LDL. Figure 4A shows that the protein abundance of ApoAI was reduced in LDL from MSPH women compared to MPH (*p* < 0.05) (Figure 4A). In addition, the levels of triglycerides and cholesterol (total, free and ester) were determined in LDL isolated from MPH and MSPH women. In LDL from MSPH, the levels of triglycerides were 43% higher compared to MPH (*p* < 0.05) (Figure 4B). No changes in cholesterol levels were determined (Figure 4C).

### 3.5. Oxidative Status of Maternal Plasma and LDL from MPH and MSPH Pregnancies

**Figure 5:**
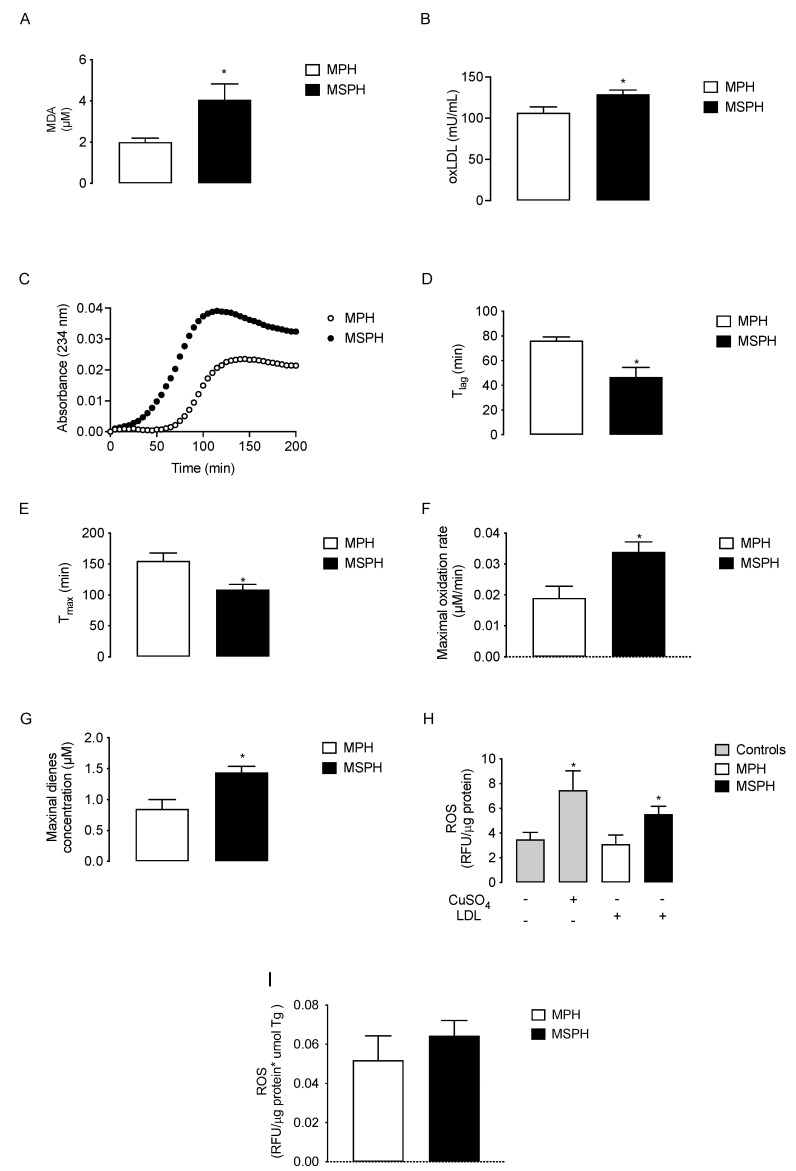
Oxidative status of maternal plasma and LDL from MPH and MSPH pregnancies. Levels of malondialdehyde (MDA, (**A**)) and oxidized LDL (oxLDL, (**B**)) determined in total serum of MPH (white) and MSPH (black) women (*n* = 12 per group). C-G, kinetic parameters obtained from conjugated dienes formation assay in LDL isolated form MPH (white) and MSPH (black) maternal serum (*n* = 10 per group): (**C**) kinetic of conjugated dienes formation; (**D**) duration of the lag-phase (T lag); (**E**) time of maximal formation of dienes (T max); (**F**) maximal concentration of dienes and (**G**) oxidation rate of LDL. (**H**,**I**) Pro-oxidant capacity of LDL from MPH and MSPH women. (**H**) Total levels of reactive oxygen species (ROS) were determined in control HUVEC (grey) or HUVEC incubated with the following: CuSO4 (gray), LDL isolated from MPH (white) or MSPH (black) maternal serum. (**I**) Levels of ROS were determined in HUVEC incubated with LDL isolated from MPH (white) or MSPH (black) maternal serum. All values were corrected by triglyceride concentration in LDLs. *n* = 7 per group. Values are mean ± S.E.M. * *p* < 0.05 versus values for MPH.

Lipid peroxidation determined as malondialdehyde generation (MDA, *n* = 12 per group) was two times higher in MSPH serum compared to MPH (*p* < 0.05) (Figure 5A). The levels of oxLDL also increased 21.6% in MSPH compared to MPH (*p* < 0.05) (Figure 5B). To determine the oxidative status of LDL, conjugated dienes formation was determined in isolated lipoprotein forms MPH and MSPH (*n* = 10 per group). The curve of kinetic formation of conjugated dienes showed higher levels of dienes for LDL from MSPH women (Figure 5C). The technical parameters evaluated from the oxidation kinetic showed that the duration of the lag-phase (T lag) and the time of maximal formation of dienes (T max) were reduced in MSPH samples compared to MPH (38.5% and 29.9%, respectively, *p* < 0.05) (Figure 5D,E). The maximal concentrations of dienes and oxidation rate were 78.4% and 70.2% higher for MSPH samples compared to MPH (*p* < 0.05) (Figure 5F,G). Finally, to determine the pro-oxidant capacity of LDL from MPH and MSPH women, the levels of ROS were determined in HUVEC incubated with the lipoproteins or CuSO_4_ as a positive control of oxidation (*n* = 7 per group). As shown in Figure 5H, the levels of ROS were 58.1% higher in cells incubated with LDL from MSPH compared to MPH (*p* < 0.05). Considering that LDL from MSPH presented increased levels of triglycerides compared to LDL from MPH, the levels of ROS were corrected by the concentration of triglycerides in LDL. As shown in Figure 5I, under this condition there was no difference between the groups.

## 4. Discussion

In this work, different parameters associated with lipid levels and oxidative status were analyzed in a group of pregnant women without pathologies or obstetrics complications. In these women, the levels of TC, LDL, triglycerides and PCSK9 increased from the first to third trimester. At T3, the women were categorized as MPH or MSPH according to its total cholesterol levels using a cut of value of 280 mg/dL, a value previously used by different groups in different reports [1,5,7,9,27]. Maternal serum levels of TC, LDL and PCSK9 as well as placental abundance of PCSK9 were higher in MPSH compared to MPH women. In addition, circulating PCSK9 levels were positively correlated with LDL at T3, especially in MPSH women. After the evaluation of lipid peroxidation and oxidized LDL levels, both parameters were shown to be higher in MPSH compared to MPH women. In LDL isolated from MSPH women, triglycerides were higher and the abundance of ApoB was higher and that of ApoAI was lower compared to MPH. The formation of conjugated dienes was earlier and higher in LDL isolated from MSPH, and endothelial cells incubated with those LDL presented increased levels of reactive oxygen species compared to LDL from MPH.

Lipid determination at T1 and T3 showed that the levels of total cholesterol, LDL and triglycerides increase as pregnancy progresses, a result consistent with data previously published [1,4,7,31]. Additionally, the levels of circulating PCSK9 increased significantly by 39.4% between the first and third trimester. A previous report showed increased levels of PCSK9 in pregnant women at delivery compared to non-pregnant women [18]; however, no studies addressing the levels of PCSK9 along pregnancy has been described previously. We propose that the described increased in PCSK9 could be related with increased levels of LDL from T1 to T3. To date, a possible role of PCSK9 as a regulator of maternal LDL levels along pregnancy has been neglected, even considering that the protein could be regulated by estrogen and insulin levels [17,32,33,34,35], hormones that increases during pregnancy [36,37]. Interestingly, the role of PCSK9 as a regulating factor of LDL circulating levels has been considered as a therapeutic target. In fact, biological inhibitors of PCSK9 are currently developed and approved to be used in hypercholesterolemic non-pregnant subjects, which have shown good results associated with the reduction in LDL and cardiovascular risks [14].

Regarding the lipid levels at terms of pregnancy in MPH and MSPH women, only total cholesterol and LDL levels increased in the MSPH group. Despite that, the lipid levels of the neonates were similar and the results are consistent with data previously published [1,5,7,9,27], and eventually, they are mediated by changes in the cholesterol traffic described in MSPH placentas [9]. To date, there is no information related with possible maternal or fetal factors leading to the supraphysiological increase in maternal lipids, even knowing that this maternal condition is associated with endothelial dysfunction of macro- and microvascular vessels of the placenta [4,7,8,28], altered traffic and content of cholesterol in placental trophoblasts [9] and the development of atherosclerosis in the offspring [1,2,10] of women with MSPH pregnancy. Interestingly, we reported for the first time a correlation between maternal levels of LDL and PCSK9, a correlation that is stronger if only MSPH women are considered. In the paper of Peticca et al., they reported no correlation between PCSK9 and LDL levels at terms of pregnancy of the studied pregnant women [18], a result that differs from ours and that could be related with the population studied. Our study only considered pregnancies without associated pathologies or metabolic alterations. In the paper of Peticca et al., 36% of women presented preeclampsia and 27% intrauterine growth restriction, and all samples were grouped [18]. Other factors to consider is that, in the aforementioned study, women with TC levels over 280 mg/dL or women that could be classified as MSPH were not included. Therefore, our results showed not only an association between maternal LDL levels and PCSK9 but also that this association is higher for MSPH women who also have increased total levels of circulating PCSK9 and increased protein levels in the placenta. The magnitude of change in circulating PCSK9 reported in our study in MSPH women (18.7%) is comparable to changes determined in other studies in non-pregnant populations that measured PCSK9 levels in multiethnic populations, including men and women. These studies described that PCSK9 increased about 11 and 17%, respectively, in patients with hypercholesterolemia [38] or with myocardial infraction [39] compared to the control subjects.

To determine if increased maternal lipid levels in women with other conditions, different from MSPH, also associate with increased levels of PCSK9, we measured the lipid and protein levels in a group of women with GDM. As shown, PCSK9 levels were not increased in these women even when the levels of TC and LDL increased, suggesting that the association between PCSK9 and LDL could be related to MSPH and not to GDM. As already pointed out, there are no studies addressing the possible mechanisms involved in the onset of MSPH; based on the described data, we suggest that PCSK9 could play a role in this phenomenon, which would ultimately be involved on the pathogenesis of the MSPH condition. Undoubtedly, this hypothesis needs to be confirmed with other groups of patients with similar characteristics. Additionally, the mechanisms associated with increased levels of PCSK9 also need to be elucidated. In this scenery, it could be feasible to speculate the possible effects of increased maternal levels of PSCK9 not only in the maternal lipid profile and vasculature but also in its effect on the offspring. It has been described that the offspring of MSPH women present fetoplacental endothelial dysfunction, arterial fatty streaks at birth and increased cardiovascular risk in adulthood [2,10]. Whether PCSK9 regulating maternal LDL levels could be also associated with those offspring outcomes needs to be determined.

The second part of this study focused on determining whether, in addition to increased PCSK9 and LDL levels, there are other pro-atherogenic characteristics in MSPH women. Therefore, by measuring MDA levels, we determined an increased oxidative status suggesting that MSPH women could be exposed to increased lipid peroxidation and oxidative stress, which had been previously proposed [27]. Following this finding, we measured the levels of oxLDL, and we found that this pro-atherogenic lipoprotein is also increased in MSPH women [40]. Interestingly, a reciprocal relationship between PCSK9, oxLDL and one of its receptors, LOX-1, has been proposed in the context of atherosclerosis in non-pregnant populations, contributing to the development of the disease [41,42,43,44]. However, it remains to be elucidated if this relationship is also relevant in the context of MSPH pregnancies, where both PCSK9 and oxLDL are elevated.

To delve into the features of maternal LDL, we determined the levels of the main LDL proteins and the triglycerides and cholesterol levels in isolated lipoproteins, determining that ApoB, ApoAI and triglycerides levels were different in isolated LDL from MPH and MSPH women. Increased levels of ApoB in LDL from MSPH women were determined in our study. As mentioned in the Introduction, ApoB synthesis increased during pregnancy because of maternal hormonal changes. In addition, it has been reported that, in small and dense LDL, the LDLs with a major proatherogenic potential also are enriched in ApoB [45,46]. Based in this information, we speculate the possibility that increased levels of ApoB in LDL from MSPH could be due to the increased synthesis of this apolipoprotein in the maternal liver and to major levels of small and dense LDL.

Even when ApoB accounts for over 95% of the LDL protein, the presence of other proteins could change its biological behavior. In LDL from MSPH women we determined reduced levels of ApoAI compared to LDL from MPH women. Unfortunately, we have no information about the possible repercussions of this change on LDL function. Despite this, it has been reported that, in electronegative and proatherogenic LDL, the levels of ApoAI are increased [47] and that LDL enriched in ApoAI could be a marker of cardiovascular risk [48]. Considering this information, the contribution of ApoAI in LDL in our study model needs to be determined.

Our results showed that LDL from MSPH women have increased triglyceride content (47%). Regarding this result, it has been described that small and dense LDL, as well as electronegative LDL, also presents increased levels of triglycerides [47,49]. Additionally, triglycerides-enriched LDL have been reported to exhibit altered lipid delivery to cells and do not regulate cholesterol biosynthesis in cells [50]. Thus, we suggest that in MSPH women, LDL presented a pro-atherogenic profile compared to LDL from MPH. 

Moreover, considering the increased levels of MDA and oxLDL in the serum of MSPH women, we assayed the oxidative status of maternal LDL from MPH and MSPH by analyzing the susceptibility to oxidation and the pro-oxidant capacity of these LDLs. For this purpose, we first determined the susceptibility to oxidation in terms of the formation of conjugated dienes, which correspond to lipid peroxidation products for which its formation can be measured continuously at 234 nm in a cell-free assay [30,51]. The kinetic curves showed that conjugated dienes were formed earlier and in greater quantity when LDLs from MSPH women were incubated with CuSO_4_ compared to MPH. In accordance, T lag and T max were reduced, while the maximal concentration of dienes and the oxidation rate increased in LDLs from MSPH compared to MPH women, being therefore more susceptible to oxidative modifications. Similar results have been reported in LDL from preeclamptic women compared to normal pregnancies [52]. Thus, considering (1) the increased levels of PCSK9 described in our MSPH group, (2) PCSK9 levels positively correlating with small and dense LDL, and (3) our results regarding the oxidation of LDL, we suggest that LDL from MSPH could be smaller and denser than those from MPH women; in adults, it has been reported that this subfraction of LDL is more susceptible to oxidation compared to larger particles [53,54]. LDLs that undergo oxidative modifications can cause endothelial damage and trigger atherosclerotic lesions [55,56]. In accordance, we showed that when endothelial cells were incubated with maternal LDL from MSPH, ROS levels increased compared to MPH, which suggest that these particles have an increased pro-oxidant potential in a cellular system. Similarly, Cominacini et al. reported that oxLDL increases the production of ROS in HUVEC [57], which could occur by a mitochondrial-dependent mechanism [58]. Interestingly, when the ROS levels were corrected by the concentration of triglycerides in the LDL from different conditions, there was no difference between the cells incubated with LDL from MPH or MSPH, suggesting that the described difference in triglycerides content in the LDLs from MSPH could be related to increased ROS production in the cells. Taken together, we suggest that LDLs from MSPH women have an impaired oxidative status, which may potentially have implications for their vascular health and is reflected in the increased levels of lipid peroxidation products and oxLDL in the serum. This may contribute to explaining several previous observations, such as the proatherogenic lipoprotein alterations found during gestation as well as the oxidative alterations found at birth.

## 5. Conclusions

Considering our findings, we conclude that lipids and PCSK9 increase along with pregnancy and that, in the group of women categorized as MSPH, the levels of TC, LDL and PCSK9 are higher than in the control or MPH group. We propose that, in MSPH women, the increased maternal levels of PCSK9 could contribute to the maternal elevated levels of LDL, which exhibits pro-atherogenic characteristics related mainly to a pro-oxidative profile. Therefore, we suggest that the profile described in MSPH women could impair the vascular health of these women, which needs to be deeply determined. Finally, we want to emphasize that the lipid profile is not commonly assessed during pregnancy or considered in clinical practice despite evidence of a possible impact of MSPH not only on placental function and vasculature of the offspring but also on maternal vasculature.

## Figures and Tables

**Table 1:** Clinical characteristics of pregnant women and their newborns.

Group	MPH-T1 (*n* = 16)	MPH-T3 (*n* = 44)	MSPH-T3 (*n* = 43)	GDM-T3 (*n* = 19)
***Maternal variables***				
Weeks of gestation for lipid determination	12.7 ± 0.4 * (12.4–13)	39.3 ± 1.7 (37–41)	39.2 ± 0.8 (37.4–41)	39.2 ± 0.8 (38–40)
Age (years)	31.5 ± 2 (32–35)	29.8 ± 4.9 (20–35)	29.3 ± 4.6 (23–37)	34.5 ± 2 * (32–35)
Height (cm)	160 ± 16 (148–172)	161.9 ± 6.5 (150–176)	162.1 ± 5.6 (150–175)	160.8 ± 6.4 (155–178)
Weight (kg)				
T1	63 ± 12.7 (52–72)	63.1 ± 7.2 (50–79)	61.1 ± 7 (46–79)	64.3 ± 2 (63–67)
T2	70 ± 14.2 (58–75)	68 ± 7.5 # (53–82)	67.3 ± 7 # (56–85)	69.3 ± 5.1 # (64–78,5)
T3	74.1 ± 12.9 (65–83)	73.7 ± 7 # (61–90)	73.9 ± 6.8 # (54–88)	72.1 ± 8.3 # (56–85)
BMI (kg/m^2^)				
T1	24.5 ± 2.3 (23–25)	24 ± 2 (19–28)	23.4 ± 2 (18–27)	24.1 ± 3.7 (20–27)
T2	25.3 ± 2.9 (24–26)	25.6 ± 2 # (19–30)	25.4 ± 2.1 # (20–28)	26.5 ± 2.4 # (22–29)
T3	28.9 ± 2.1 # (28–30)	27.7 ± 2 # (23–30)	28.1 ± 2 # (24–30)	27.8 ± 3.2 # (22–33)
Systolic arterial pressure (mm Hg)				
T1	105 ± 4 (100–110)	109 ± 7 (100–120)	102.7 ± 9 (84–120)	113.3 * ± 5.8 (110–120)
T2	105 ± 4.4 (100–110)	107 ± 10 (90–130)	104.4 ± 9.8 (90–130)	115 * ± 13.8 (100–130)
T3	109 ± 5.9 (98–100)	110.4 ± 9.1 (90–130)	110.7 ± 10 (90–128)	114.3 ±13.1 (100–137)
Diastolic arterial pressure (mm Hg)				
T1	65 ± 7 (60–70)	69.2 ± 9 (60–70)	63.5 ± 8.6 (60–80)	69 ± 12.8 (55–80)
T2	63 ± 5.2 (60–70)	68.5 ± 8 (53–88)	63 ± 4.6 (60–70)	67.7 ± 5.8 (52–70)
T3	64 ± 4.9 (60–80)	68.8 ± 8 (58–89)	68 ± 7 (50–80)	68.9 ± 8.6 (60–85)
Basal glicemia T1 (mg/dL)	82.5 ± 10.6 (75–90)	79 ± 7.2 (68–100)	80.7 ± 6.7 (64–91)	83.7 ± 13.6 (66–115)
OGTT (mg/dL) T2				
Basal glycaemia	73.5 ± 0.7 (71–90)	76.6 ± 6.6 (67–22)	76.3 ± 5.3 (68–87)	76.3 ± 4.7 (69–83)
Glycaemia 2 hrs. after glucose	94 ± 6.3 (79–105)	104 ± 28 (64–130)	95.5 ± 16 (63–120)	151.7 ± 10 * (142–173)
***Newborn variables***				
Sex (female/male)	10/6	20/24	23/20	10/9
Gestational age (weeks)	38.7 ± 1 (38–40)	39.3 ± 1.7 (37–41)	39.2 ± 0.8 (37.4–41)	39.2 ± 0.8 (38–40)
Birth weight (g)	3362 ± 280 (2700–4250)	3308 ± 316 (2830–4210)	3318 ± 422 (2660–4250)	3254± 488 (2560–3890)
Height (cm)	49.5 ± 2.1 (47–53)	50.5 ± 1.5 (47–53)	50.3 ± 1.7 (47–56)	49.4± 2.7 (47- 53)
Ponderal index (g/cm^3^ × 100)	2.7 ± 0.1 (2.2–2.8)	2.6 ± 0.2 (2.2–3)	2.7 ± 0.2 (2.2–3.1)	2.7± 0.2 (2.5–2.9)
APGAR score 1 min.	8.7 ± 1 (6–9)	8.5 ± 1.1 (5–9)	8.5 ± 1.1 (5–9)	8.4 ± 1.3 (6–9)
APGAR score 5 min.	9 ± 0.3 (8–10)	8.9 ± 0.5 (8–10)	9.1 ± 0.5 (8–10)	8.8 ± 1.1 (6–9)
Lipid levels in umbilical blood (mg/dL)		*n* = 16	*n* = 12	*n* = 12
Total cholesterol	-	60.6 ± 12.1 (46–77)	63.6 ± 14.4 (47–97)	69 ± 27.3 (40,118)
HDL	-	30.7 ± 7.7 (20–44)	29.4 ± 7.5 (22–48)	25.8 ± 11.8 (15–46)
LDL	-	22.9 ± 5.7 (16–35)	26.2 ± 8.3 (13–37)	30.3 ± 15.7 (19–64)
Triglycerides	-	35.5 ± 17 (21–80)	39.6 ± 14.8 (20–66)	39 ± 27.3 (26–69)

Women with maternal physiological (MPH, TC ≤ 280 mg/dL) or supraphysiological hypercholesterolemia (MSPH, TC > 280 mg/dL) at delivery were included (see Methods). Weight, body mass index (BMI) and blood pressure were determined at trimester (T) 1, 2 and 3. Lipid profiles were determined at T1 or T3 as indicated. BMI, body mass index. OGTT, oral glucose tolerance test. APGAR, Appearance, Pulse, Grimace, Activity and Respiration. HDL, high-density lipoprotein; LDL, low-density lipoprotein. *: *p* < 0.05, versus corresponding values in the other groups. #: *p* < 0.05 versus corresponding values for the parameter in the same group at T1. Data are presented as mean ± S.D. (range).

## Data Availability

Data is contained within the article.
